# High energy ball milling as a strategy to improve electrochemical performance in sodium titanate anodes

**DOI:** 10.1039/d6ra04923k

**Published:** 2026-10-28

**Authors:** Pooja Kumari, Yining Li, Rebecca Boston

**Affiliations:** a School of Chemical, Materials and Biological Engineering, University of Sheffield Sheffield S1 3JD UK poojaphysics417@gmail.com R.boston@sheffield.ac.uk

## Abstract

High energy ball milling has emerged as a method for both mechanosynthesis and post processing to control the particle size and crystallinity in Li and Na ion battery materials. Sequentially higher ball milling speeds were applied to commercially available Na_2_Ti_3_O_7_ as a post-processing method, followed by chemical and electrochemical characterisation. At lower milling speeds, the Na_2_Ti_3_O_7_ phase was preserved, but with some degradation of crystallinity and phase. At higher milling speeds, full amorphitisation occurred, followed by recrystallisation into the rarely-observed trigonal Na_4_Ti_5_O_12_ phase. The sequential changes in electrochemical properties of the materials following increasing ball mill speeds are reported, with the partially degraded Na_2_Ti_3_O_7_ showing increases in specific capacity which we attribute to a decrease in crystallite size and associated access to kinetically limited sites. Trigonal Na_4_Ti_5_O_12_ by contrast, is measured to have superior rate capability (40 mA h g^−1^ at 5C), and high cycle stability, alongside a promising specific capacity of 115 mA h g^−1^ after 50 cycles. This material is therefore a promising anode candidate for Na-ion batteries.

## Abstract 2

Improvements observed in specific capacity and cycle stability following high energy ball milling: Na_2_Ti_3_O_7_ transforms into Na_4_Ti_5_O_12_.

## Graphical abstract

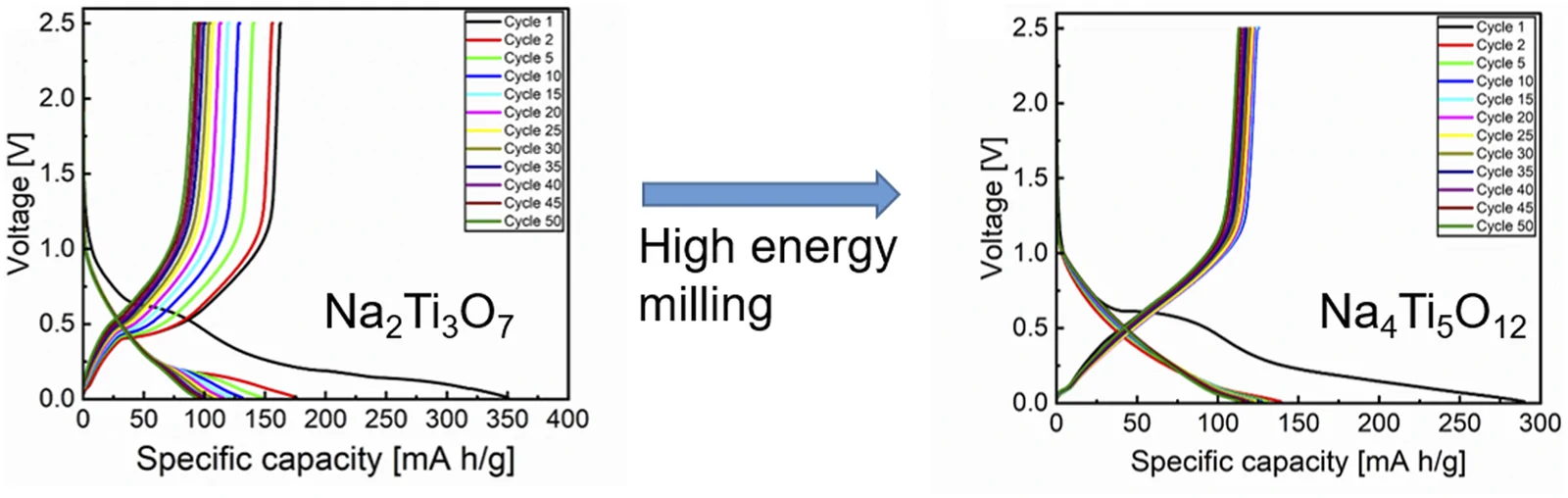

## Introduction

Sodium ion batteries represent a viable option to supplement rechargeable battery technologies as the demand for scarce Li increases beyond available resources.^1^ Na for use in batteries have a number of promising characteristics, including chemistry similar to that of Li, high abundance and wide global distribution.^2^ Significant successes have been achieved in layered Na-ion cathode materials using a direct transfer of chemistry from Li-ion technologies; challenges remain however in developing anodes for Na-ion use. Here, a similar transfer of materials or approach from Li-ion technologies has shown poor performance. Graphite is a prime example of this, with non-graphitic or hard carbons have been extensively studied, with typical values of reversible capacity >300 mA h g^−1^ (0.1–0 V *vs.* Na^+^/Na), close to that for Li insertion in graphitic carbon.^2^ There are limitations for hard carbons in terms of their practical use, as they suffer from electroplating and poor cyclability^3^ which leads to safety concerns. Also, poor capacity retention and low electrolyte compatibility means it is still necessary to explore alternative anode materials for Na-ion batteries.^4^

One such alternative is the sodium titanate family of materials, which exist in a variety of compositions and crystal structures. Layered (*e.g.* Na_2_Ti_3_O_7_) and tunnel structure (*e.g.* Na_2_Ti_6_O_13_ or Na_4_Ti_5_O_12_) sodium titanates are of particular interest, although neither in isolation satisfy the requirements for commercial cells. Whilst Na_2_Ti_6_O_13_ has high stability, it does not show a high enough specific capacity to be of significant interest (theoretical capacity *c*. 50 mA h g^−1^. Similarly, the tunnel structured Na_4_Ti_5_O_12_ in either its monoclinic^5^ and low temperature trigonal^6^ forms has also been shown to have low capacity (*c*. 50 mA h g^−1^), due in part to the steric hindrance of the large (*vs.* Li) Na ion, in direct contrast to the superior performance of Li_4_Ti_5_O_12_.^7^

Na_2_Ti_3_O_7_, in contrast, is considered as a potential anode material for Na-ion batteries because of its high theoretical capacity of 177 mA h g^−1^ (at 1C current density), and low activation energy for Na (de)insertion (around at 0.3 V). It has a layered structure, characterised by zig-zagged edge- and corner-sharing TiO_6_ octahedra, forming sheets between which the Na ions can intercalate.^8^ leading to favourable performance across multiple performance indicators.^9^ Na_2_Ti_3_O_7_ suffers with intrinsic low electronic conductivity and structural distortion through movement of Na^+^, so is by no means a final usable composition.^10^ Furthermore, repetitive volume expansion and contraction during battery (de)intercalation leads to structural degradation. In our previous work,^11^ it was clear that Na_2_Ti_3_O_7_ electrodes suffered from more irreversible loss, shorter cycle life, and rapid capacity fading than other members of the sodium titanate family (*e.g. vs.* the conversion process of Na_2_Ti_6_O_13_), despite its high theoretical and early cycle capacity. To accommodate these issues, various efforts have been made to improve performance, including nanostructured electrode materials such as nanoparticles, nanowires, nanotubes, or carbon composites with Na_2_Ti_3_O_7_.^12–14^ Nanoscaled materials hold significant promise due to the higher surface area, which decreases the overpotential and permits faster reaction kinetics at the electrode surface.^15^ This is tensioned, however, against increased interaction with the electrolyte, leading to greater solid electrolyte interphase (SEI) formation and associated degradation.^16^

To prepare these nanostructured electrode materials, a number of synthesis methods have been trialled *e.g.* hydrothermal,^17^ solvothermal,^18^ solid state reaction,^19^ and sol–gel.^20^ In other areas of Li and Na electrode synthesis, high energy ball milling has started to gain traction as a route to synthesise nanoscale materials and/or novel crystal structures.^21^

High energy ball milling is a means by which large amounts of energy can be transferred to materials to induce reactions without the need for high temperatures.^21^ On the laboratory scale, this consists of small milling vessels (*e.g.* useful capacity of 30 mL) containing spherical milling media composed of ceramic or metals, which are rotated in a way to create radial centrifugal force.^22^ This transfers energy during milling through collisions, creating repeated impacts and compression forces and shear. This must be balanced against critical speeds, where the rotational speed is so great as the physically pin the milling media against the wall of the milling pot, resulting cessation of any milling action.^23^

The transfer of energy can induce a number of processes including fracturing (resulting in reduction of particle size to the point of amorphitisation), and cold welding or mechanical alloying.^24^ The amorphitisation–cold welding process is of particular interest as it creates an environment where reactions which would otherwise require high temperatures to occur, and potentially access phases which would not form at high temperatures. As such, the technique has been used across a wide range of materials.^25,26^

High energy ball milling has been used for a limited number of sodium titanates,^27–29^ for example composite Na_2_Ti_6_O_13_–Na_4_Ti_5_O_12_ was synthesised using high energy ball milling in combination with a heating step between 300 and 100 °C, forming a range of multiphase materials.^27^

On the other hand, use of high energy ball milling as post-synthesis powder processing, where the goal has been used to reduce particle size and to introduce partial amorphitisation, is not well studied in Na-ion materials. The smaller particle size can benefit to decrease the Na diffusion length and contribute to improved rate capabilities,^30^ as have been measured in, for example Mn-doped titanate nanowires.^30^ Nano-sized Na_2_Ti_3_O_7_ has also been shown to provide improved performance at high discharge rates and low temperatures, due to the kinetics of the electrode materials that is significantly dependent on the particle size.^31–33^ In these reports, the reduced particle size plays a critical role in the reversibility.^34,35^ Thus, methods that can reduce the particle size of electrode materials are of significant interest to improve the performance of the electrode. There is also the possibility that at higher energies, crystal structures may decompose and materials may change composition *via* a transient amorphous phase, or may enable access to metastable phases.

Challenges still remain, therefore, in optimising the performance of sodium titanate materials. While mechanosynthesis (from reagent materials) is one potential route, post-synthesis processing may also represent an opportunity to reduce particle size (and correspondingly increase surface area), increase degree of amorphitisation, and potentially access metastable phases which cannot form *via* high temperature routes.^29^

Herein, we seek to understand how high energy ball milling can be used to improve electrochemical performance in sodium titanates. Commercially available Na_2_Ti_3_O_7_ was ball milled at sequentially higher speeds, with phase, physical and electrochemical properties evaluated.

## Methods

### Sample preparation and characterisation

Na_2_Ti_3_O_7_ was purchased from Sigma-Aldrich (UK) and used without further purification. Na_2_Ti_3_O_7_ was milled under an inert Ar atmosphere, using a Fritsch Premium Line 7 Planetary (P7) ball mill, for up to 10 h at the following speeds: 200 rpm (NTO-200); 400 rpm (NTO-400); 600 rpm (NTO-600); and 800 rpm (NTO-800). Milling pots were loaded with approximately 1 g of sample into 45 mL silicon nitride grinding bowls with 20 mL useful capacity (steel casing) containing 20 zirconium oxide grinding balls, *Ø* 5 mm. The milling pots were of the standard geometry provided with the P7, comprising a shallow (depth = 12 mm; diameter 45 mm) dish, with a flat lid. For all mechanochemical reactions, data analysis was performed *ex situ*. After milling, samples were transferred and stored in a vacuum oven at 80 °C to prevent water ingress.

X-ray diffraction (XRD) measurements were performed using a PANalytical X'pert^3^ powder diffractometers, both using Ni-filtered Cu Kα radiation (*λ* = 1.5406). Phase analysis for all samples was performed using High Score (PANalytical) software.

Single crystalline domain size, *t*, was calculated using the Debye–Scherrer relationship by measuring the full width at half maximum (*θ*_B_) of the peaks measured in XRD, eqn (1):

1 *t* = *Kλ*/*β* cos *θ*_B_

where *λ* is the wavelength of the X-rays used (Cu K_α_ in this case, 1.540589 Å), *β* the line broadening factor, and a shape factor *K*, of 0.9 which is generally used for spherical particles. This is a reasonable assumption given the SEM.

An Inspect F50 SEM (FEI) instrument was used to investigate particle morphology. Samples for SEM were prepared by affixing powders on double-sided carbon sticky tape and sputter-coated with a thin layer of gold.

### Coin cell preparation

Half cells were prepared with sodium metal as counter electrode. A mass ratio of 7 : 2 : 1 of active Na_2_Ti_3_O_7_ material, carbon black Timical Super C65, and polyvinylidene fluoride (PVDF) were used for anode slurry preparation. Carbon black and PVDF were purchased from MTI Corp (USA) and used as a conductive agent and binder in the slurry mixture respectively. To dissolve the PVDF binder, a sufficient volume of *N*-methyl-2-pyrrolidone (NMP, anhydrous 99.5%, Sigma-Aldrich) was used as a solvent, followed by mixing in a Thinky ARE-250 mixer. Slurries were coated onto carbon-coated aluminium foil (thickness *c*. 250 µm) using a micrometre adaptable doctor blade, followed by drying at 80 °C for 24 h. Calendaring was performed using a rolling mill (MTI, MSK-HRP-MR100DC), decreasing the electrode thickness to ensure close contact between the current collector and material. A disk of coated foil *Ø* 12 mm (area = 1.13 cm^2^) was prepared using an electrode cutter for use in coin cell fabrication.

Coin cell assembly was performed in an argon filled glove box (MBraun, O_2_ & H_2_O <0.5 ppm) using the prepared disks, GF/F Whatman glass microfiber filter separator, and sodium metal (sodium ingot, 99.8%, Alfa Aesar) as the counter electrode. A solution of 1 M NaPF_6_, dissolved in 1 : 1 ethylene carbonate and diethyl carbonate solvent was used as an electrolyte (Kishida).

### Electrochemical measurements

Coin cells were cycled at different current densities, using a cut-off voltage window of 0.01–2.5 V (*vs.* Na^+^/Na) in a Neware CT-4008T 5 V 10 mA battery cycler in an ECO HYCL900 temperature and humidity-controlled chamber set to 25 °C. Cyclic voltammetry (CV) measurements were conducted using a Maccor series 4300 battery cycler between cut-off voltages of 0.0 V–2.5 V (*vs.* Na^+^/Na) at a scan rate of 0.08 mV s^−1^.

All electrochemical measurements were performed on at least three cells, with representative datasets presented herein.

## Results and discussion

Powder XRD was used to determine the crystal structure and phase of all samples as shown in Fig. 1. The as-received powder NTO-0, Fig. 1, indicates that the sample is predominantly composed of monoclinic Na_2_Ti_3_O_7_ (*P*2_1_/*m*) (PDF card 00-031-3129) with a minor secondary phase of monoclinic Na_2_Ti_6_O_13_ (*C*2_1_/*m*) (PDF card 01-091-7833).

**Fig. 1:**
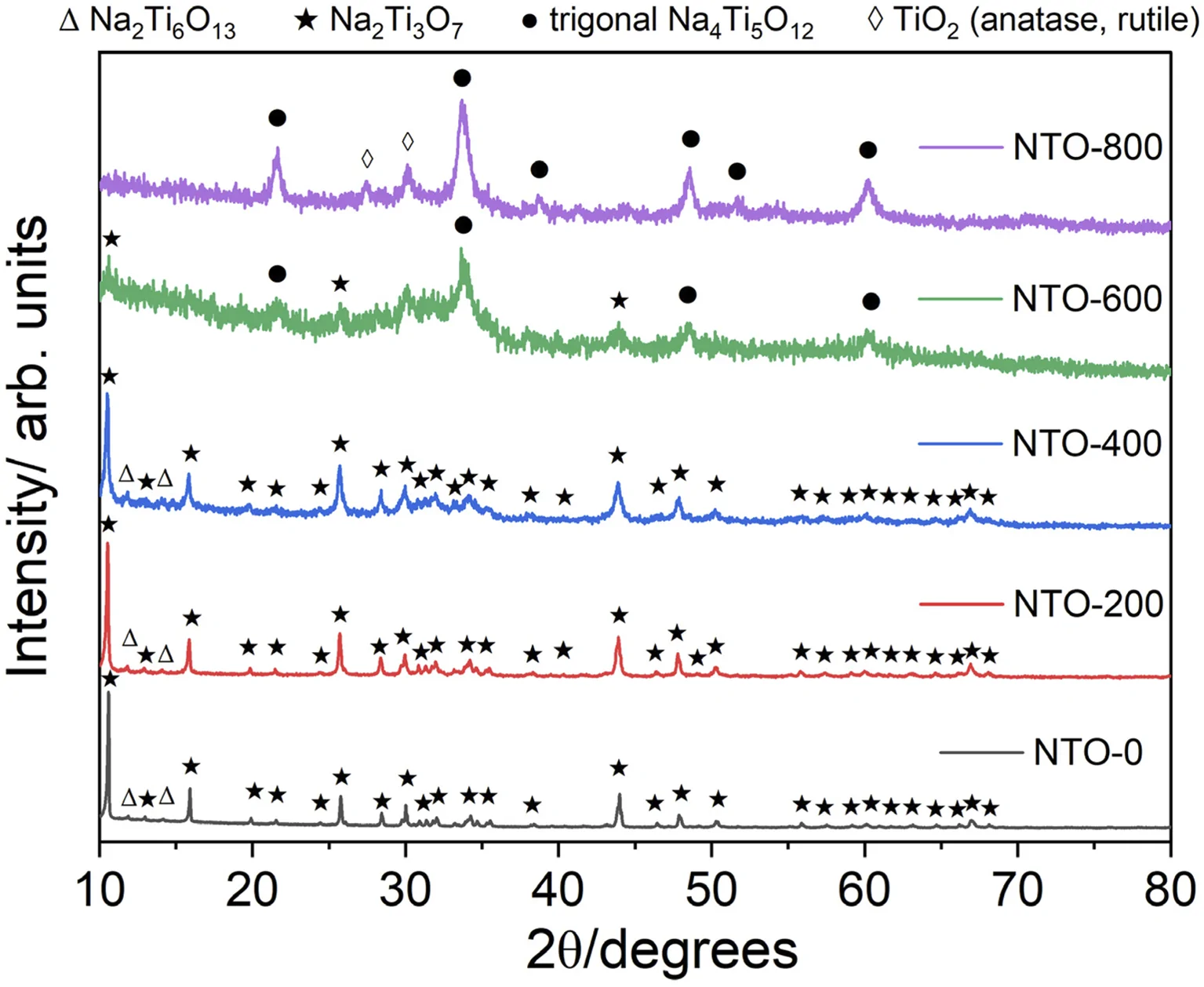
XRD patterns of the as-received material NTO-0 composed primarily of Na_2_Ti_3_O_7_, followed by milling for 10 h at the following speeds: NTO-200 milled at 200 rpm, NTO-400 milled at 400 rpm, NTO-600 milled at 600 rpm, and NTO-800 milled at 800 rpm. The lower speed milling indicates some degradation of crystal structure, before total re-organisation from 600 rpm onwards into the Na_4_Ti_5_O_12_ phase.

Fig. 1 also shows the XRD patterns for samples ball milled for 10 h at 200, 400, 600 and 800 rpm respectively. NTO-200 shows a very similar pattern to the as-received material, and indicates little loss of crystallinity. As previously, the phases can be indexed to a monoclinic Na_2_Ti_3_O_7_ phase and monoclinic Na_2_Ti_6_O_13_. These phases are also present after milling at 400 rpm, although there is an appreciable amount of peak broadening which indicates that the single crystalline domain size is decreasing.^36^

When milling speed was increased further (NTO-600), clear differences *vs.* the as-received powder were observed. Significant amorphisation occurred, with little of the original two phases remaining. Alongside a very small amount of Na_2_Ti_3_O_7_, peaks corresponding to the low temperature trigonal Na_4_Ti_5_O_12_ (P3, PDF card 04-017-0817) start to emerge. These are broad, and relatively weak peaks, indicating small areas of the long-range order required to be considered crystalline. After milling at 800 rpm, Na_4_Ti_5_O_12_ is the only sodium titanate phase present. A minor anatase TiO_2_ phase (PDF card 00-021-1272) is also observed, forming as a result of the sodium titanate phase losing Ti. Here again there is significant peak broadening *vs.* the as-received powder, suggesting that there has been total destruction of the initial crystal structure, and emergence of the new phase through mechanosynthesis. The trigonal Na_4_Ti_5_O_12_ phase has not been extensively studied as an anode for Na-ion batteries, as it has previously been shown to have only modest electrochemical performance *vs.* other members of the sodium titanate family.^6^

Scanning Electron Microscopy (SEM) was conducted on the samples to investigate morphology and particle size. The as-received powder (Fig. 2(a)) shows the typical mixture of rod-like and particulate structures commonly seen in Na_2_Ti_3_O_7_. All of the ball milling regimes tested here destroyed this morphology, as seen in the images in Fig. 2(b)–(e), with particle shapes becoming less well defined with the higher milling speeds. Fig. 2(d) and (e) don't indicate any further reduction in particle size, however when considered alongside the XRD in Fig. 1, it is clear that the evident particle size from SEM is no longer representative of the single crystalline domain size.

**Fig. 2:**
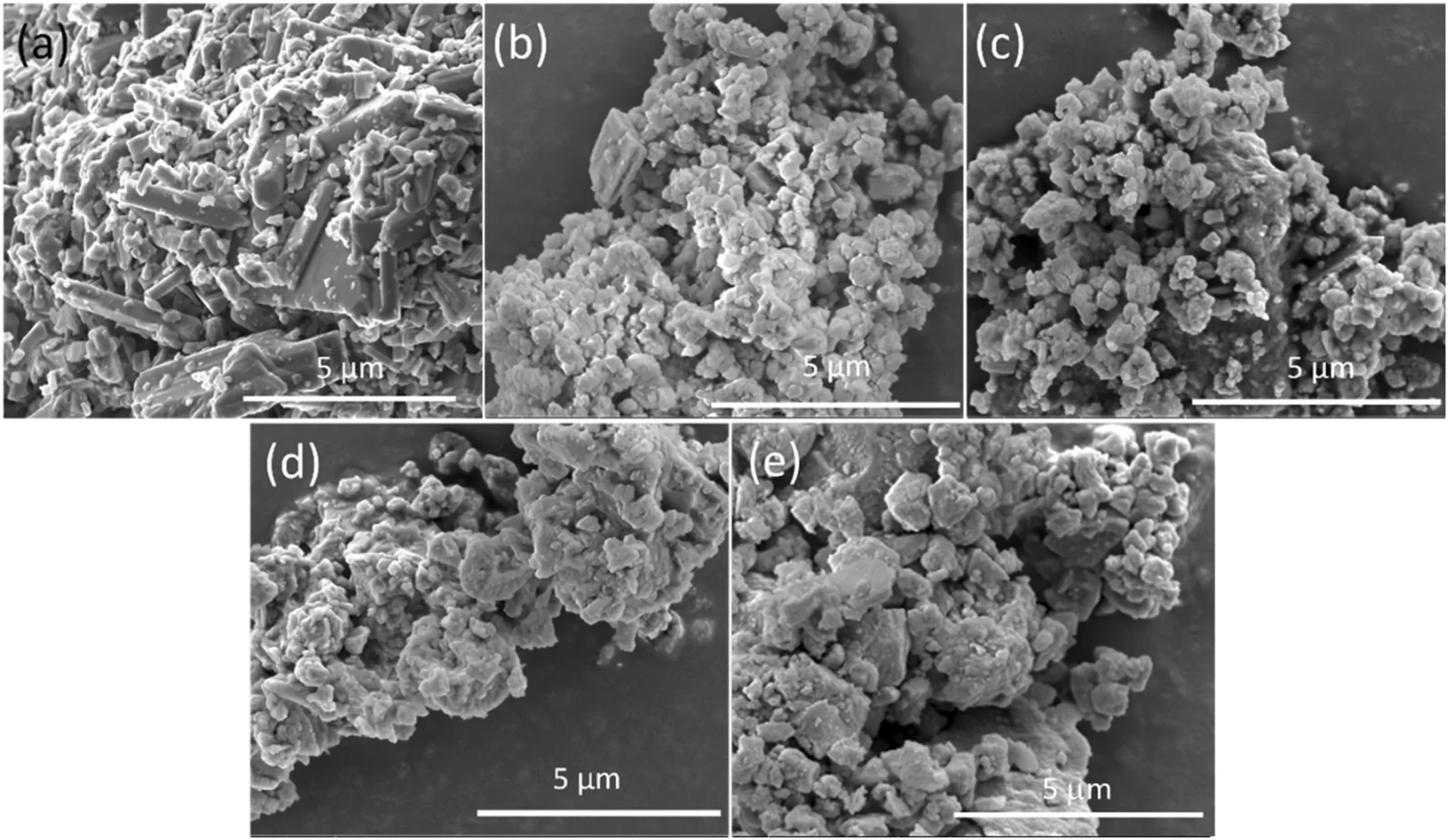
SEM images of (a) the as-received powder NTO-0, (b) NTO-200, (c) NTO-400, (d) NTO-600, (e) NTO-800. The partially rod-like morphologies disappear with even low energy milling, and by NTO-400, the particle size is decoupled from single crystalline domain sizes.

To confirm this, Debye–Scherrer analysis was performed on NTO-0, NTO-200, NTO-400 and NTO-800, to quantify the size of the smallest single crystalline domains. These are shown in Table 1. Analysis was not attempted on NTO-600 due to the low counts and low signal-to-noise (Fig. 1).

**Table 1:** Comparison of calculated single crystalline domain sizes using eqn (1)

Sample	Calculated domain size/nm
NTO-0	63 ± 10
NTO-200	46 ± 15
NTO-400	25 ± 7
NTO-800	14 ± 3

This clearly demonstrates the decoupling of the single crystalline domain size from the apparent particle size (SEM). There is no discernible change in particle size between NTO-200 and NTO-800 (and intervening samples), however analysis of the XRD peak breadth indicates that the single crystalline domain size reduces for faster milling times.

Half cells of all samples were prepared for electrochemical testing. Cyclic voltammetry (CV) measurements were performed to investigate the redox couples of all five NTO electrodes (0.08 mV s^−1^ scan rate, voltage window 0 V–2.5 V), as shown in Fig. 3 (a)–(e). Fig. 3 (a) shows the CV curves of the as-received NTO (NTO-0) for the first 5 cycles, with redox peaks at 0.01 V and 0.33 V, which are related to the Ti^4+^/Ti^3+^ redox couple.^37,38^ The first cathodic scan (Na+ ion insertion) shows a side reaction of electrolyte reduction started at 1.2 V and another wide peak at approximately 0.3 V. This corresponds to the formation of solid electrolyte interphase (SEI) film, occurring between the electrode materials and electrolyte.^9^ It can be speculated that the SEI film formation is essentially complete in the first cycle,^39^ as this peak is not observed in subsequent cycles.

**Fig. 3:**
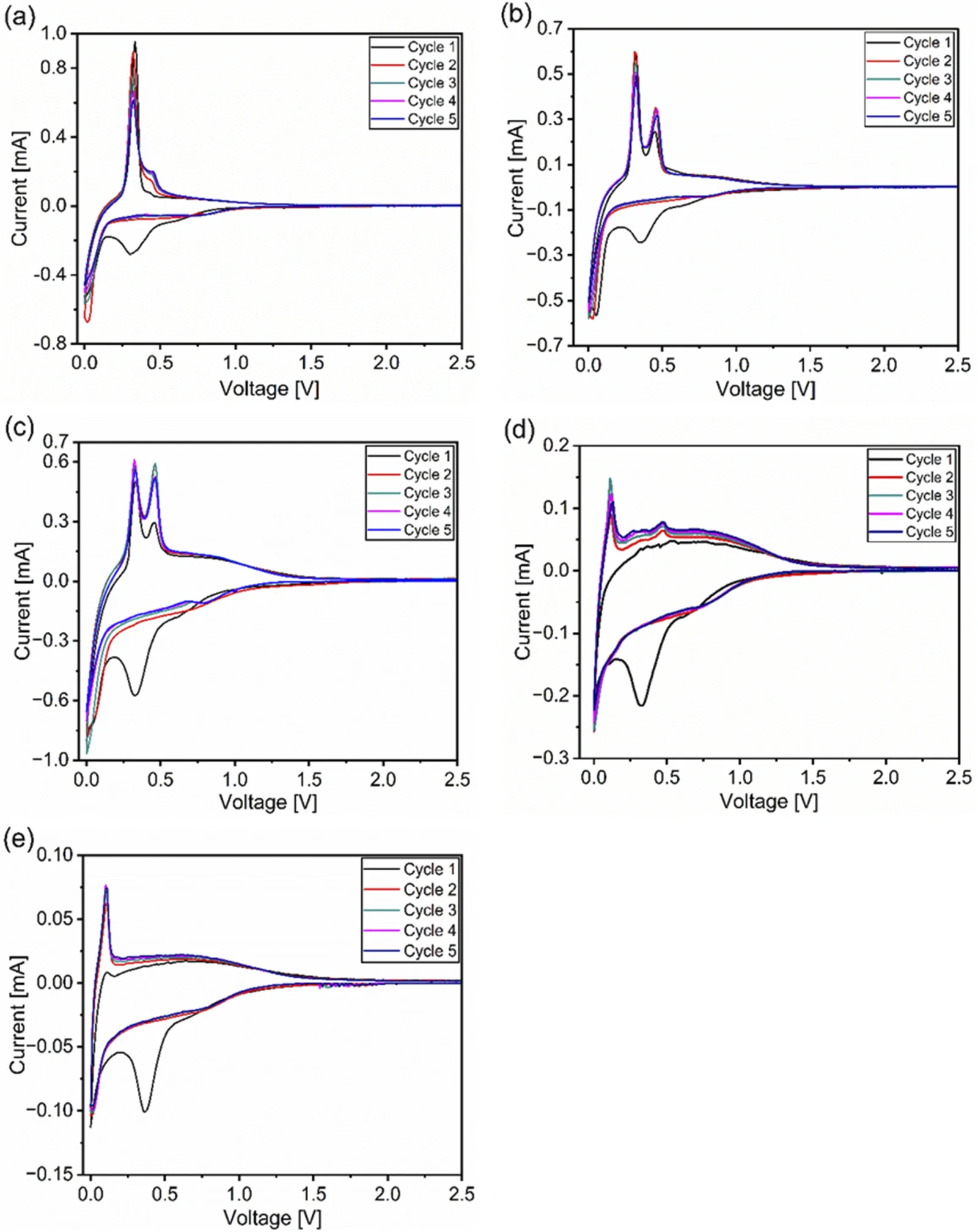
Cyclic voltammograms for all samples (a) NTO-0, (b) NTO-200, (c) NTO-400, (d) NTO-600, (e) NTO-800, showing a distinct change in electrochemical processes between the samples containing predominantly Na_2_Ti_3_O_7_ ((a)–(c)) and Na_4_Ti_5_O_12_ ((d)–(e)).

During the first anodic scan, a prominent peak at 0.33 V with a shoulder peak at 0.43 V was observed, corresponding to the two known extraction voltages of Na+ from Na_2_Ti_3_O_7_.^9^ A decrease in the current density of the peak at 0.33 V is observed as cycle number is increased, attributed to changes in structure as cycles progress.^40^ The peak at 0.43 V appears from the second cycle onwards, although remains small throughout, compared to the lower voltage peak. In previous work, this peak has been attributed to a kinetically limited process,^41^ and its low current density here suggests that the larger particle size (and lower surface area) in this sample hindering Na extraction at this voltage.

Similar peak positions are recorded for NTO-200 (Fig. 3(b)) and NTO-400 (Fig. 3(c)), correlating with the similarities in the phases identified by XRD (Fig. 1). The peak at 0.33 V in NTO-200 and NTO-400 (Fig. 3(b) and (c)) shows a smaller overall current density from the first cycle *vs.* the as-received powder NTO-0, however the two ball milled samples show smaller changes in subsequent cycles, and stabilise at a higher overall current density than NTO-0. This extraction process is thought to be thermodynamically limited, and impacted by structural changes during cycling.^41^ As such, we attribute the higher peak stability and overall larger current density (at 5 cycles) to reduced structural degradation in the ball milled samples. It is likely that what would otherwise be cycle-induced degradation has already occurred during the milling process.

The peak at 0.43 V in both NTO-200 and NTO-400 (Fig. 3(b) and (c)) shows an increasing magnitude with increasing milling speed *vs.* NTO-0. Hwang *et al.* posit that the Na ion extraction from this site which produces this peak is kinetically limited during cycling.^40^ The observations made here suggest that higher rpm ball milling improves Na extraction kinetics for this sodium site, the smaller single crystalline domain size enabling higher Na ion mobility.

These two observations should result in higher specific capacity in NTO-200 and NTO-400 *vs.* the as-received powder, after initial capacity fade. It might also result in poorer performance of NTO-400 at higher cycling rates, as the kinetically limited process at 0.43 V plays a larger role in the capacity of this sample. In all cases, electrochemical impedance spectroscopy would be needed to confirm these hypotheses.

As indicated by the XRD in Fig. 1, NTO-600 and NTO-800 are composed of a primarily of Na_4_Ti_5_O_12_, although this is indicated as highly nanoscale and not well crystallised, particularly the NTO-600 sample, along with some TiO_2_ and amorphous material. This is formed during total pulverisation of the original material to an amorphous bulk, followed by recrystallization of the new phases, see eqn (2). The low crystallinity makes quantitative analysis (*e.g.* Rietveld refinement) highly unreliable, and therefore deconvoluting the exact active masses and potential contribution of the amorphous phases extremely challenging. The amorphous contribution in NTO-800 is minimal (if present at all) and so if we assume full conversion of the Na_2_Ti_3_O_7_ to Na_4_Ti_5_O_12_, this should result in one mole of TiO_2_ for every mole of Na_4_Ti_5_O_12_, a maximum TiO_2_ mass fraction of 13%.

2 2Na_2_Ti_3_O_7_ → Na_4_Ti_5_O_12_ + TiO_2_

This is reflected in the CV curves, Fig. 3(d) and (e) for NTO-600 and NTO-800 respectively, which show a markedly different peak profile compared to the Na_2_Ti_3_O_7_ samples. For first cathodic sweep of NTO-600 sample (Fig. 3(d)), there are three reductive peaks, located at 0.32 V, a small peak at 0.11 V, and at 0.01 V respectively. The peak at 0.32 V in both of these samples again corresponds to the SEI formation disappears in the following cycles, following the same pattern as all of the other samples.

In the anodic sweeps, the first cycle shows a small amount of signal from the remaining Na_2_Ti_3_O_7_ between 0.25 V and 0.46 V, and is likely to correspond to the same electrochemical processes as observed in the other samples. This feature has a much smaller current density than in the samples which have undergone less milling, and indicates that the phase change identified by XRD in NTO-600 is not fully complete, with some of the original Na_2_Ti_3_O_7_ material remaining.

After the first cycle, this small feature persists, and a new feature at 0.15 V develops. This we attribute to the Na_4_Ti_5_O_12_ phase, showing more electrochemical activity than previous research, due to the wider voltage window used. Woo *et al.* used a window from 0.3 V upwards, and therefore miss the process which we identify here.^6^ This explains the low specific capacity they observed, *vs.* the higher capacities we measure.

The single anodic peak in NTO-800 (Fig. 3(e)) indicates that the material has been fully converted from Na_2_Ti_3_O_7_ to Na_4_Ti_5_O_12_, corroborated by XRD (Fig. 1), which shows a better crystallised phase *vs.* NTO-600, and clearer peaks from the residual TiO_2_.

It is clear from the CV that there are two distinct materials present depending on degree of milling: the original Na_2_Ti_3_O_7_ up to 400 rpm, with changes in behaviour relating to the availability of the two Na sites; and nanoscale trigonal Na_4_Ti_5_O_12_ above 400 rpm, which shows a corresponding shift in the electrochemical processes in the cell.

The electrochemical battery performance of commercially available Na_2_Ti_3_O_7_, and ball milled samples was investigated by galvanostatic charge/discharge measurements at 25 °C with a potential window of 0.01–2.5 V. Fig. 4(a) shows the specific capacity for all samples over the first 50 cycles and it is clear that all samples exhibit good cycle stability over the period measured.

**Fig. 4:**
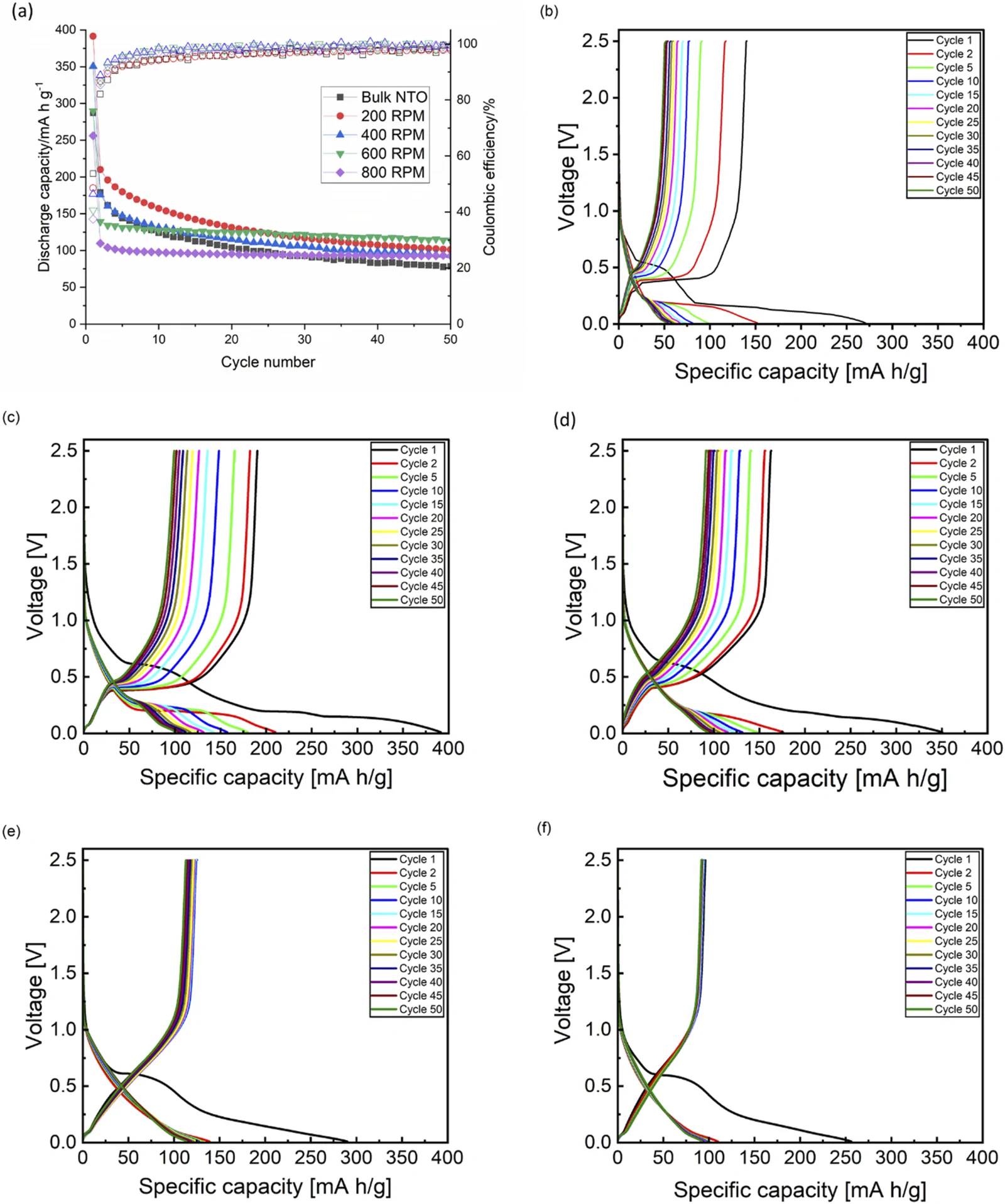
(a) Summary of specific discharge capacities up to 50 cycles at 0.05C. Individual charge and discharge profiles for each sample are shown in (b) NTO-0, (c) NTO-200, (d) NTO-400, (e) NTO-600, and (f) NTO-800. The compositional differences identified within the samples are reflected here in the different profiles.

All samples were subject to first cycle losses due to SEI formation. This was also observed in the CV (Fig. 3) and summarised in Table 2. All samples initially perform very similarly to exemplar Na_2_Ti_3_O_7_ materials, showing the characteristic first cycle losses. At 50 cycles, however the NTO-600 and NTO-800 samples (Na_4_Ti_5_O_12_) show superior capacity retention. Hard carbon still, however, leads the field for capacity and retention thereof.

**Table 2:** Comparison of first and second cycle capacities and initial coulombic efficiency. First and second cycle capacities and coulombic efficiencies are given for 0.05 C unless stated otherwise

Sample	First cycle specific capacity/mA h g^−1^	Second cycle specific capacity/mA h g^−1^	Initial coulombic efficiency/%	50th cycle specific capacity/mA h g^−1^	Reference
NTO-0	270	151	56	76	This work
NTO-200	392	210	54	100	This work
NTO-400	350	176	50	115	This work
NTO-600	290	139	48	130	This work
NTO-800	252	130	52	100	This work
Na_2_Ti_3_O_7_	300 (0.1C)	205 (0.1C)	66 (0.1C)	70 (0.1C)	42
Mixed sodium titanates	330	167	51	91	43
Hard carbon	310 (0.1C)	295 (0.1C)	95 (0.1C)	290 (0.1C)	44

All ball milled samples had higher remaining specific capacity after 50 cycles than the as-received material. At 50 cycles at 0.05C the as-received material had a specific capacity of 76 mA h g^−1^, *vs.* NTO-200 with a specific capacity of 100 mA h g^−1^ and NTO-400 with 115 mA h g^−1^.

The (dis)charge profiles for NTO-0, NTO-200 and NTO-400 (Fig. 4(b)–(d) respectively) all show similar features, in line with the XRD and CV results. Several plateaux are observed in the first discharge, however only the plateau at 0.2 V is observed in later cycles. Interestingly, despite the differences in relative peak heights for the two extraction processes observed in Fig. 3(b) and (c), this is not reflected in the overall specific capacity of the material. Both NTO-200 and NTO-400 show very similar specific capacity after 50 cycles. The shape of the profiles is indicative of intercalation behaviour, as is well known for Na_2_Ti_3_O_7_.^41^

Following the milling-induced phase change, NTO-600 and NTO-800 show highly stable behaviour, following first cycle losses, stabilising at 130 mA h g^−1^ and 100 mA h g^−1^ respectively at the second cycle, Fig. 4(e) and (f). Indeed, NTO-600 shows the highest specific capacity after 50 cycles of all the materials tested in this study. This shows that the trigonal Na_4_Ti_5_O_12_ phase formed by the higher energy ball milling strategy is a promising Na-ion anode material in its own right. For these two samples the profiles take a different shape, with no obvious plateaux. This is more indicative of conversion-like behaviour, in line with the type of electrochemical behaviour of tunnel structure materials.^17^

The rate performance of the as-received NTO-0 and ball milled NTO samples is shown in Fig. 5. Fig. 5(a)–(c) shows the rate performance of NTO-0, NTO-200 and NTO-400, all identified as Na_2_Ti_3_O_7_ with successive amounts of disturbance to the crystal structure. NTO-200 (Fig. 5(b)) mirrors the observed increased specific capacities, and retains better rate capability at 5C than NTO-0. This is likely to be due to a favourable combination of factors: the smaller particle size enables easier Na ion extraction at higher rates from the 0.3 V site, with a contribution from the 0.46 V site. In NTO-400, the high rate capacity drops to almost zero, which we attribute to the dominance of the 0.46 V kinetically limited site: even with the smaller single crystalline domain size, the high rate means that there is not enough time for these kinetic processes to occur. This is supported by the recovery of capacity at 0.05C following the 5C cycles, indicating that the battery is still functioning well, but that the processes cannot occur at 5C.

**Fig. 5:**
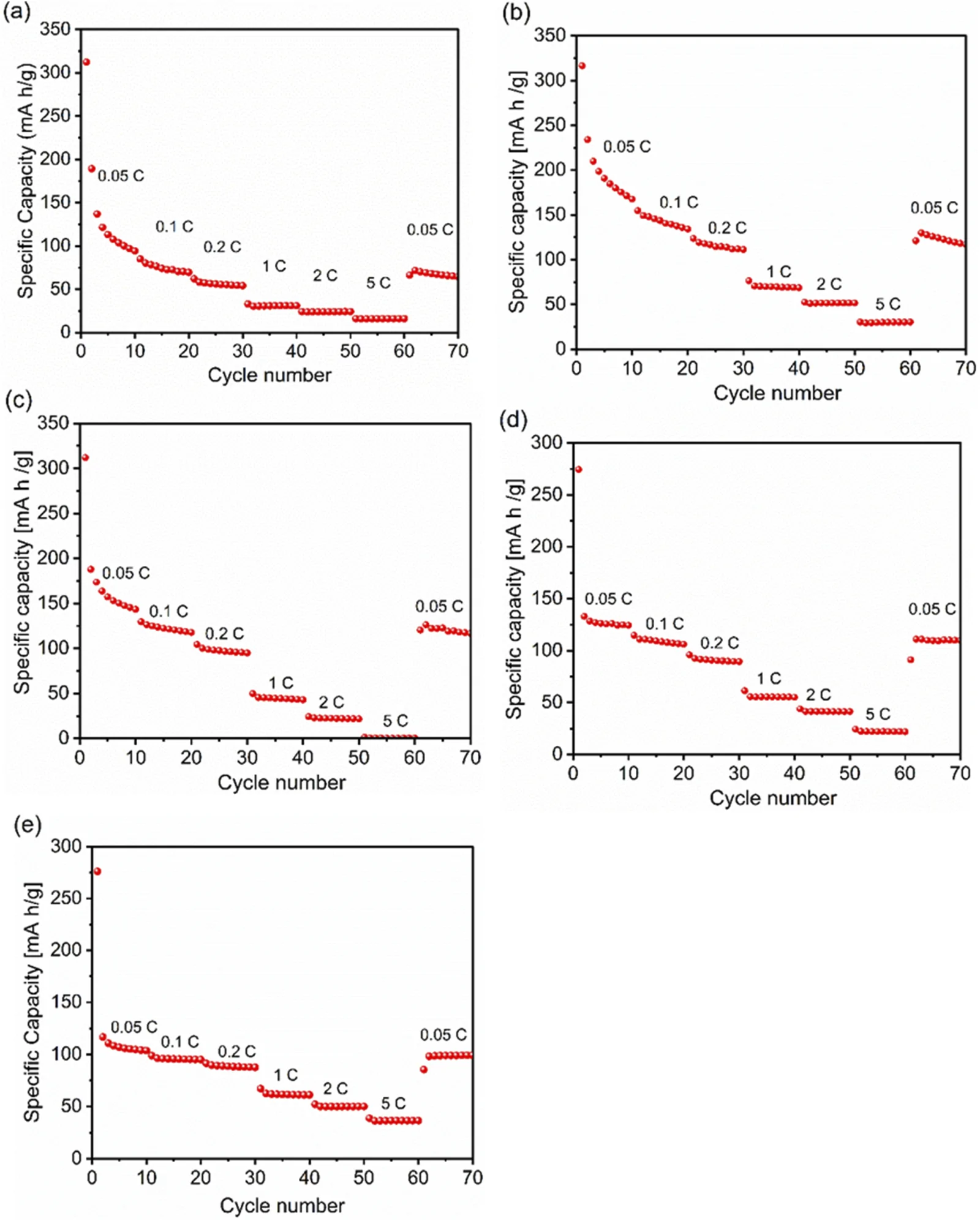
Rate capability plots for (a) NTO-0, (b) NTO-200, (c) NTO-400, (d) NTO-600, and (e) NTO-800, showing superior performance at high *C*-rate for the Na_4_Ti_5_O_12_ compositions.

The Na_4_Ti_5_O_12_ phase in NTO-600 and NTO-800, Fig. 5(d) and (e) respectively, show good rate performance, particularly NTO-800 at 5C, which shows a capacity of 40 mA h g^−1^, a superior rate for any NTO material. This further corroborates the hypothesis that the material formed by milling shows conversion behaviour.

The mechanosynthesised trigonal Na_4_Ti_5_O_12_ shows considerable promise as a highly stable and high rate capability material. We attribute this to the single, conversion-type process which occurs during cycling, which avoids the damaging structural changes seen in intercalation materials. Whilst using decomposed Na_2_Ti_3_O_7_ isn't the most efficient method to do so, and results in TiO_2_ impurities which reduces the volume of active material in the electrode, it enables initial study of the trigonal form; further work is required to establish if this phase can be synthesised as a phase pure form, and whether this results in further improvements to electrochemical performance.

## Conclusion

In conclusion, we have demonstrated that high energy ball milling of Na_2_Ti_3_O_7_ can be used to both improve the electrochemical performance of the extant material, and be used as a mechanosynthesis route to form nanoscale trigonal Na_4_Ti_5_O_12_. Milling Na_2_Ti_3_O_7_ for a short time can be used to both improve the anode specific capacity and rate capability, through accessing more of the kinetically limited sites in the material, and relying less on the lower voltage site which is known to suffer from structural degradation. Tapping into this effect is limited, however, as when the kinetic processes start to dominate, the higher rate capabilities start to fail.

By further increasing the milling speed, a new material was formed: the low temperature trigonal phase of Na_4_Ti_5_O_12_. This nanoscale material was demonstrated to have both promising cycle stability and specific capacity (130 mA h g^−1^ after SEI formation and first cycle losses), and excellent rate capability at 5C (40 mA h g^−1^). This shows that the trigonal Na_4_Ti_5_O_12_ phase, which has previously been dismissed as low-capacity, benefits strongly from nanoscale crystalline domains and emerges as a promising material for use in sodium ion batteries.

## Conflicts of interest

There are no conflicts to declare.

## Data Availability

Processed data is uploaded to the University of Sheffield Online Research Data Repository (ORDA) database. It is currently under embargo which will be lifted if the associated manuscript is accepted for publication. ORDA follows FAIR principles. Once de-embargoed this can be accessed at https://doi.org/10.15131/shef.data.31872796, with the summary available now.
